# Comparison of blood pool thresholding to manual contouring of the right ventricle in patients with suspected ARVC

**DOI:** 10.1186/1532-429X-17-S1-P305

**Published:** 2015-02-03

**Authors:** Stephen Lyen, Jonathan C Rodrigues, Helen Mathias, Mark Hamilton, Nathan E Manghat

**Affiliations:** 1 Queen Elizabeth Hospital, Birmingham, UK; 2 Bristol Heart Institute, Bristol, UK

## Background

Right ventricular (RV) dilatation is a key imaging criterion of the 2010 modified task force criteria for the diagnosis of arrhythmogenic right ventricular cardiomyopathy/dysplasia (ARVC/D), which makes accurate and reproducible volumetric anaylsis essential. We wanted to evaluate the effect of using new thresholding software in volume analysis compared to standard manual contouring on reproducibility and accuracy.

## Methods

Retrospective RV volume analysis was performed on 20 anonymised cases of suspected ARVC. 8mm contiguous short-axis cine steady-state free precession (SSFP) data was acquired from the base of the ventricles to the apex on a 1.5 Tesla MRI scanner. Volumetric analysis was then analysed using two different commercially available softwares: (1) Semi-automated blood pool thresholding with CVI42 (Circle Cardiovascular Imaging, Alberta) and (2) Manual contouring with Argus Syngo (Siemens Medical Systems, Germany). Independent analysis was performed by four different experienced observers, two for each software. RV papilla and trabeculations were excluded from the blood volume. Indexed RV end diastolic volume (IEDV), end systolic volume (IESV) and ejection fraction (EF) were recorded. Statistical analysis included Bland-Altman plots and intraclass correlation coefficients (ICC) to assess for accuracy and interobserver variability.

## Results

There was excellent interobserver agreement in RV volumes for both blood pool thresholding (IEDV ICC: 0.93, IESV ICC: 0.93, EF ICC: 0.88) and manual contouring (IEDV ICC: 0.90, IESV ICC: 0.96, EF: 0.95), with no statistically significant difference between each set of two observers (p>0.05).

Whilst there was good correlation between indexed volumes between the two methods (IEDV r^2^=0.93, IESV r^2^=0.95), Bland-Altman analysis demonstrated significant mean bias (IEDV mean bias = -13ml/m^2^, Limits of agreement -23.6 to -3.6ml/m^2^; IESV mean bias = -8.7ml/m^2^, Limits of agreement -19.6 to 2.2ml/m^2^). The thresholding values were consistently lower than the manual contouring.

The average contouring time with thresholding was slightly shorter at 190 seconds compared to 262 seconds for manual contouring.

## Conclusions

The significant mean bias between thresholding and manual contouring raises important questions about the reliability between different tools we use to perform volume analysis. The bias may due to enhanced detection of more subtle trabeculations in the RV wall which would normally be excluded manually by the observer. Partial voluming occurs at the interface of the endocardium and blood pool, which may cause thresholding software to underestimate volumes. In addition, it is unclear if the software used itself may contribute to this bias. This is of particular importance in ARVC where there are currently clearly defined thresholds for abnormal RV volumes, however these factors should be considered whenever instutions upgrade software, or when comparisons are made to reference values performed with specific software.

## Funding

This research was supported by NIHR Bristol Cardiovascular Biomedical Research Unit.

**Figure 1:**
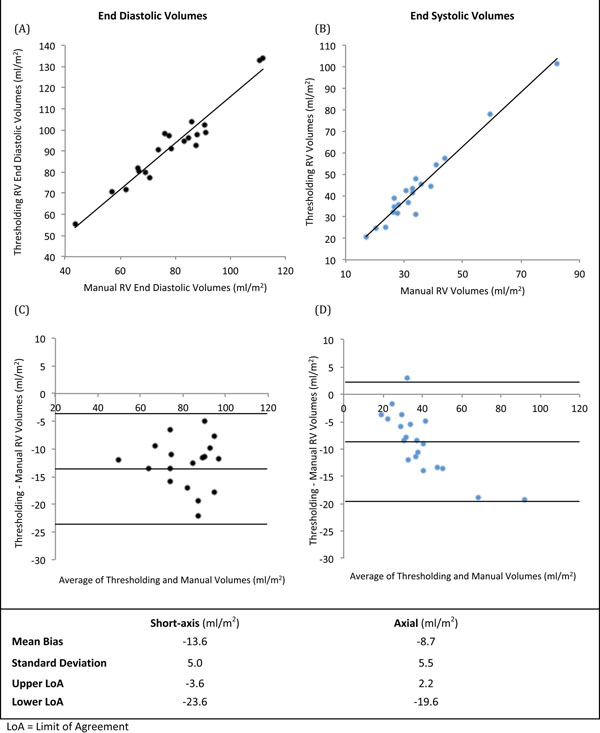
Comparison of thresholding and manual RV volumes.

